# Dynamics of Chinese Diet Divergence from Chinese Food Pagoda and Its Association with Adiposity and Influential Factors: 2004–2011

**DOI:** 10.3390/ijerph17020507

**Published:** 2020-01-13

**Authors:** Jiajun Zhou, Sirimaporn Leepromrath, Xu Tian, De Zhou

**Affiliations:** College of Economics and Management, China Center for Food Security Studies, Nanjing Agricultural University, No. 1, Weigang, Xuanwu District, Nanjing 210095, China; zhoujiajun_06@163.com (J.Z.); lougmoo@outlook.com (S.L.); xutian@njau.edu.cn (X.T.)

**Keywords:** dietary patterns, diet quality index, nutrition transition, China

## Abstract

Nutrition transition in China has a strong impact on dietary quality and health of Chinese consumers. This study developed the diet quality divergence Index (DQD), the divergence between real food consumption and the Chinese food pagoda 2016 (CFP), to measure the quality of diet in China. Using four waves of data (2004, 2006, 2009, and 2011) from China Health and Nutrition Survey (CHNS), this study shed light on the transition of diet quality for Chinese residents. Results indicate that the DQD generally decreased and Chinese diet quality improved during 2004–2011. The divergence was mainly caused by over-consumption of legumes and nuts, and under-consumption of milk and milk products. Rising income and urbanization were positively correlated with diet quality for the people with low DQD. However, both of them had negative impacts on diet quality for those with high DQD. Females and rural residents held a lower DQD than their counterparts. The results also revealed that healthy food preference, education, dining at home, household size, proportions of teens (6–17) and elders (over 64) in the families are positively correlated with Chinese diet quality. However, labor intensity, frequency of drinking alcohol, and smoking have negative impacts on diet quality. Moreover, higher DQD was found to be associated with increasing risks of overweight/obesity. Therefore, we suggest national healthy policies should pay more attention to nutrition education. It is also necessary to focus on populations with poor diet quality and to adopt measures to control drinking alcohol and smoking.

## 1. Introduction

Chinese have been experiencing a remarkable nutrition transition with the rapid economic growth in the past decades [1,2,3,4,5]. The diet patterns gradually shift from the traditional diet, which is dominated by cereals and vegetables, towards the pattern associated with high intake of fat and calorie density foods. The nutrition transition has strong impacts on the national health, as one emerging economy, China encounters both obesity and malnutrition problems [4,6,7]. In the past two decades, the number of overweight and obese people in China raised rapidly [8]. By 2018, China had the largest number of obese people all over the world, including 43.2 million males and 46.4 million females, respectively [9]. A large number of studies pointed out that diet quality is significantly associated with a range of noninfectious chronic diseases caused by over-nutrition, such as obesity, diabetes, hypertension, coronary heart diseases, and certain types of cancers [10,11,12,13,14,15,16]. However, the transition of Chinese diet quality is still not clear enough. Therefore, to deepen the understanding the Chinese diet quality transition would have strong policy implications for the national nutrition and health policies and food demand prediction in China.

Diet quality is one hot topic in researches on food demand and many studies paid attention to diet quality measurement in developed countries, such as healthy eating index (HEI) [17,18,19,20,21,22], diet quality index (DQI) [10,23], Mediterranean diet scale (MDS) [24,25,26] and their derivatives. Only a few studies provided measurements of diet quality in China. According to ‘Chinese Dietary Guidelines 1997’, Stookey et al. [27] proposed the INFH-UNC-CH DQI to measure the Chinese diet quality without revealing the dynamics of Chinese diet transition. There are some studies focused on certain aspects of diet quality (e.g., dietary patterns, dietary diversity), or the dietary quality for specific groups (e.g., children, the elderly) or relationships between dietary quality and certain diseases [4,5,7,28,29,30,31,32,33,34]. Recently, some studies used INFH-UNC-CH DQI [1] and Chinese food pagoda score (CFPS) [2] to analyze the diet quality transition and its important influential factors in China.

Taking all those diet quality measurements into consideration, most of them take account of the information on food attributes, diet habits, and other attributes of diet quality, but one concern associated with those measurements is that the cut-off weights for different food items in the process of index composing are mainly designed by the researchers themselves and may be influenced by subjective choices, and this would undermine the reliability of the diet quality evaluation. In addition, most current measurements involve nutrients rather than specific food items and that may be one big challenge for researchers and policy makers to compose such kind diet quality indexes and for consumers to employ those indexes [32]. To make a complement to the diet quality measurements, this study proposes one diet quality measurement—diet quality divergence index (DQD) which is composed with the cumulative absolute divergence between the food intakes and recommendations from ‘Chinese Food Pagoda 2016’ (CFP 2016). With the data on Chinese diet, the present study tries to shed light on the transition of diet quality for Chinese household in the past decade. One advantage of the DQD is that the scoring items and food categories are derived from CFP 2016 objectively. Given there are still no authoritative and clear conclusions indicating which food category is more important than the others for diet quality, and each food category has unique attributes for health which are indispensable for a balanced diet, it is reasonable to take the equal weighted food categories in the DQD composing process [7]. DQD is also more easily handled than other diet quality measures as it takes the food items into consideration instead of nutrients. That makes it easy to apply and understand for both policy makers and consumers.

Due to the complexity of food consumption, systematic research on the influential factors of diet quality is still scant [1]. Generally, the relationships between the diet quality index for Chinese households and its influential factors are assumed to be linear in the previous studies [1,28]. However, some studies have pointed out that the impacts of some factors (e.g., income, urbanization) on diet quality are heterogeneous for different population groups [5,35,36,37,38]. Therefore, to systematically explore the impacts of the main influential factors on diet quality for different population groups with the use of quantile regressions is another target of the present study.

## 2. Materials and Methods 

### 2.1. Study Subjects 

Four waves (2004, 2006, 2009, and 2011) of China Health and Nutrition Survey (CHNS) longitudinal secondary data were employed in the present research. The CHNS is jointly implemented by the Carolina Population Center at the University of North Carolina at Chapel Hill and the National Institute for Nutrition and Health under Chinese Center for Disease Control and Prevention. The CHNS is an ongoing tracking survey of approximately 4000 families and 12,000 individuals per wave covering both urban and rural regions in nine provinces (Guangxi, Guizhou, Henan, Heilongjiang, Hubei, Hunan, Jiangsu, Liaoning, and Shandong) in China before 2011, and three autonomous cities (Beijing, Chongqing, and Shanghai) were added in 2011. A multistage, random cluster process was used to select samples in each province in the survey. Counties were stratified by income (low, middle, and high) in provinces at the beginning, and a weighted sampling scheme was used to randomly select four counties in each province. In addition, the provincial capital and a city with low income were selected when feasible. Villages and townships within the selected counties and urban and suburban neighborhoods within the selected cities were selected randomly.

The CHNS collected the weighed food consumption for each family member by using a 24 h recall method for three consecutive days in one week. The socioeconomic and demographic information such as region, household income, family size, age, gender, education, etc., are also collected in the survey [39,40].

**Figure 1:**
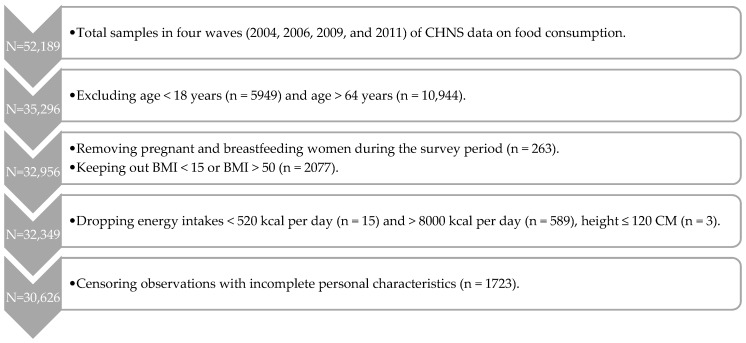
The process of sample selection.

Figure 1 shows the selection process of samples derived from the CHNS survey. There were 52,189 observed samples in total in the four waves of CHNS data on food consumption. As diet recommendations for either elders (older than 64) or children/teenagers (younger than 18) vary from those for general adults (age 18–64) in Chinese dietary guidelines 2016 (CDG 2016), this study focused on the majority populations of Chinese adults aged 18–64 years. Respondents younger than 18 years (n = 5949) or older than 64 years (n = 10,944) were excluded in the present study. Furthermore, pregnant and breastfeeding women (n = 263) were removed due to these populations also having different diet recommendations in CDG 2016. Taking the representative of the data into consideration, observations (n = 2077) with abnormal body mass index (BMI < 15 or BMI > 50) were pruned away to keep the representative of our diet quality index for normal adults. We further dropped the samples with unrealistic energy intakes which was lower than 520 kcal per day (minimum energy required for survival, n = 15) and greater than 8000 kcal per day (about four times as much calorie intake as mean, n = 589). In addition, individuals (n = 3) who are below 120 cm in height and generally considered to be patients with human short stature (dwarfism, Laron syndrome, and idiopathic short stature) were excluded, as the previous studies imply that human short stature may have a different metabolism and diet demand compared with normal adults [41,42,43]. Observations with incomplete personal characteristics (n = 1723) were also censored. Finally, 30,626 individuals from the four waves of CHNS data were employed in the present study. 

It should bear in mind that the data in this study were unbalanced longitudinal data. We got 7139, 6894, 7260, and 9333 individuals in 2004, 2006, 2009, and 2011, respectively. Compared to data in 2004, 4902 individuals were followed up in 2006. In addition, 4492 respondents were both surveyed in 2006 and 2009. Finally, for those observed in the 2009 survey, there were 4973 respondents left in 2011. Over the period of 2004–2011, only 2567 individuals were surveyed in all four waves in this study.

### 2.2. Chinese Food Pagoda 2016

Chinese dietary guidelines 2016 (CDG 2016) is a guideline for Chinese healthy diet which was jointly conducted by the Chinese Center for Disease Control and Prevention (CDC), National Health and Family Planning Commission of the People’s Republic of China, and the Chinese Nutrition Society (CNS) [44]. The Chinese food pagoda 2016 (CFP 2016) (Table 1) succinctly shows the daily recommended intakes of eight food categories for general adults (age 18–64) (Table 1): (1) Cereal and potatoes; (2) fruits; (3) vegetables; (4) eggs; (5) aquatic products; (6) meat and poultry; (7) legumes and nuts; (8) milk and milk products. The specific dietary guidelines for infants, children, adolescents, pregnant women, breastfeeding women, and old people are also presented in the CDG 2016, but not in the CFP 2016. The CFP 2016 presents a fixed amount of milk and dairy products intake, and the recommended ranges for the rest of the food categories. 

Although the recommended daily intakes of salt and oil are also mentioned in Chinese food pagoda 2016 (CFP 2016) they were not included in the present study. CHNS recorded the changes of inventory as the consumption of salt and oil rather than following the traditional 24 h recall method, meanwhile, there are no standard recipes for cooking and the use of salt and oil in the cooking process would vary across households, in addition, the waste of dish and soup is quite popular for many Chinese households and the number of dishes consumed by each family member is also ambiguous, all those together make it hard to obtain the exact individual intakes of salt and oil according to the changes of inventory [45]. Therefore, the present study did not take salt and oil into consideration.

### 2.3. Assessment of Food Consumption

CHNS collected the individual daily weighed food records (in grams) by using a 24 h recall method, and implemented them for three consecutive days in one week, including all food items the family members consumed at home and away from home. All food consumption data were recorded by trained interviewers through face-to-face structured interviews by using food pictures and models, including ingredient code, amounts, meal locations (e.g., “at home, school, restaurant, etc.”), preparation method (e.g., “boiled, stir-fried, steamed, etc.”), and preparation location, etc., of all consumed food items in each meal (i.e., breakfast, lunch, dinner) [28]. In the survey, this step was achieved by asking individuals (aged 12 years or older) each day to report all food consumed away from home during the 24 h of the previous day, and the same interview has been used to collect individual food consumption at home. For children younger than 12, the mother or a mother substitute who handles food preparation in the household was asked to recall the children’s food consumption [45]. Respondents were prompted about snacks and shared dishes. Food items consumed at restaurants, canteens, and other locations away from home were systematically recorded. Housewives and other household members were encouraged to provide additional information by which the interviewer can record the amounts of food items in dishes consumed at home.

All field workers were well trained nutritionists who are otherwise professionally engaged in nutrition work and who have participated in other national surveys. Almost all interviewers graduated from post-secondary schools and many of them have four year degrees. In addition, three days of specific training in dietary data collection was also provided by CHNS. In addition to individual dietary intakes, household food consumption in the same three consecutive days was also collected with the method of examining changes in food inventories between the beginning and end of each day. It was carried out with a combination of weighing and measuring technique and Chinese balances with a maximum limit of 15 kg and a minimum of 20 g. All leftover processed foods from the last meal before the initiation of the survey were weighed and recorded. All purchases, home production, and processed snack foods were also recorded. Whenever foods were brought in the household, they were weighed, and preparation waste (e.g., spoiled rice, discarded foods) was estimated when weighing was not possible. At the end of the survey, all remaining foods were again weighed and recorded. The records of both household and individual dietary intakes were used to check the quality of data collection. Thus, the average daily dietary intake for one individual calculated with the household survey, has been compared with his or her dietary intake based on 24 h recall data. When significant discrepancies were found, the household and the individual were revisited and asked about their food consumption to resolve discrepancies [46].

It should be noted that the food items were coded with the China Food Composition Table 2002/2004 (CFCT 2002/2004) (Table 1) in 2004, 2006, 2009, and 2011 CHNS surveys. Subsequently, in this paper, to obtain the individual average of daily consumption of each food category, the consumptions of corresponding food items were summed up and then divided by three (three consecutive days) in each survey (2004, 2006, 2009, and 2011). Detailed information about the CHNS data can be found in the previous literature [28,39,40,45,46]. 

### 2.4. Composition of the DQD

Firstly, the average daily consumptions of eight food categories for each individual were calculated according to CFP 2016 and CFCT 2002/2004 (Equation (1)). Then, for each observation, the absolute divergence value (percent) between the average daily consumption and CFP 2016 for each food category was computed (Equation (2)). Finally, the total DQD for each respondent was obtained by summing up all the divergences for eight food categories (Equation (3)). The range of DQD is [0,+∞], and one individual that gets a small DQD index indicates his/her diet quality is good and vice versa. When DQD comes to 0, it means the respondent’s diet is fully consistent with CFP 2016.

(1) $$X_{itk}=\frac{1}{3}\sum_{d=1}^{3}x_{itkd}$$

(2) $${DQD}_{itk}=\frac{\left(\left|X_{itk}-R_{k}\right|\right)}{R_{k}}\times 100\%$$

(3) $${DQD}_{it}=\sum_{k=1}^{8}{DQD}_{itk}$$

where $x_{itkd}$ is the consumption of food category k for respondent i on day d in year t, $X_{itk}$ is the average daily consumption of food category k for i respondent in year t, $R_{k}$ is the daily recommended intake of food category k in CFP 2016. ${DQD}_{itk}$ denotes the divergence between the average daily consumption of food category k and the corresponding recommendation in CFP 2016; and ${DQD}_{it}$ denotes the total divergence of eight food categories. Given the $R_{k}$ are intervals for some food categories, when $X_{itk}<min\left(R_{k}\right),\;R_{k}=min\left(R_{k}\right)$, $X_{itk}>max\left(R_{k}\right),R_{k}=max\left(R_{k}\right),$ and $min\left(R_{k}\right)<X_{itk}<max\left(R_{k}\right),{DQD}_{itk}=0$. 

### 2.5. Measurement of Obesity

Height and weight were measured directly by well-trained health workers based on a standard protocol recommended by the World Health Organization [28]. Body mass index (BMI), which is defined as the weight (kg)/square of the height (m^2^), is widely used to measure general obesity for adults [4]. For Chinese, based on the recommendations from working group on obesity in China (WGOC), BMI was divided into four categorical levels, namely underweight (BMI < 18.5 kg·m^−2^), normal (18.5 kg·m^−2^ ≤ BMI < 24 kg·m^−2^), overweight (24 kg·m^−2^ ≤ BMI < 28 kg·m^−2^), and obesity (BMI ≥ 28 kg·m^−2^) [4,47].

### 2.6. Measurement of Covariates

Detailed demographic, lifestyle, labor force participation, physical activity, and inactivity data for each individual were all collected by well-trained interviewers through face-to-face structured interviews [46]. According to the previous studies [1,2,28,34,48,49], many factors have strong impacts on the diet, including personal characteristics, income, age, gender, intensity of labor, food preferences, education, drinking, smoking, exercises time, and sedentary time, etc., the present study also takes account these variables in the analysis of Chinese diet quality. In this study, per capita annual net income is calculated by dividing the annual total household income by the number of family members and deflated by consumer price index (CPI) at 2015 prices. The study takes the completed years of formal education in the school as individual education. 

Labor intensity level is recorded according to the type of occupation in CHNS (1 = very light physical activity, working in a sitting position such as office workers; 2 = light physical activity, working in a standing position such as salesperson, laboratory technician, teacher; 3 = moderate physical activity, e.g., student, driver, electrician, metal worker; 4 = heavy physical activity, including farmer, dancer, steel worker, athlete; 5 = very heavy physical activity, such as loader, logger, miner, stonecutter). 

The paper composes food preference index, the sum of preferences for five food categories, including fruits, vegetables, fast food (e.g., KFC, pizza, hamburgers, etc.), salty snack foods (potato chips, pretzels, etc.), and sugared drinks, to measure the healthy food preference. In the survey, the respondents were asked to indicate their food preference for five food groups on a five-point Likert scale, where 1 represented “dislike very much” and 5 denoted “like very much”. Generally, fruits and vegetables are considered as healthy food [50], and we sum the answers directly for those two food groups. On the contrary, because of fast food, salty snack foods and sugared drinks are generally taken as unhealthy food [51,52], we inverted the answers, where 1 denoted “like very much” and 5 denoted “dislike very much”, and summed them up. The final food preference index ranges from 5 to 25 and the higher preference index indicates a healthier food preference. 

The number of household members and visitors has been recorded by the interviewers in each meal. The average proportion of dining at home is employed to control the dining habit. The drinking is measured with the frequency of drinking alcohol (1 = no drinking; 2 = no more than once a month; 3 = once or twice a month; 4 = once or twice a week; 5 = 3–4 times a week; 6 = almost every day). Smoking denotes the number of cigarettes per day, which is recorded in CHNS by asking each adult “how many cigarettes do you smoke per day”. The time for exercises (e.g., running, basketball, badminton, gymnastics, etc.) was recorded by asking “how much time (minutes) do you spend in a typical day during Monday–Friday, and how much (minutes) during Saturday–Sunday”. To obtain the average daily exercise time respondent spent within one week (Monday to Sunday), the exercise time in these two periods were summed up and divided by two in this study. Similarly, the time spent for sedentary activities (watching TV, video games, etc.) were collected by asking the participant “how much time (minutes) do you spend in a typical day during Monday–Friday, and how much (minutes) during Saturday–Sunday?” Following the same way of exercise time, we got the individual average daily sedentary time within one week.

When it comes to household characteristics, household size and age structure which have strong influences on food demand are adopted in this research. The proportions of teens aged 6–17 and elders over 64 in the families are used to control the heterogeneity of the household structure. To control the influential factors at community level [34], urbanization and dummy for urban/rural area are also employed in this study. Urbanization is measured by a multidimensional urbanization index, which captures the population density, physical, social, cultural, economic environment, and total 12 factors [53].

### 2.7. Statistical Methods

Firstly, the average daily DQD for the selected samples was calculated and the changes of DQD between 2004 and 2011 were reported. In order to check the rationality of DQD, the correlation between DQD and BMI was analyzed by a two-sample *t*-test. To explore the diet quality between different resident groups, the DQD for different groups were compared by graphics with respect to factors including income, labor intensity, education, food preference, proportion of dining at home, gender, drinking, smoking, household size, urbanization. In order to analyze the influential factors of DQD, a multivariate ordinary least squares regression was employed. Furthermore, quantile regressions were employed to further explore the heterogeneity of DQD. The data in this study were unbalanced longitudinal data, and some data were obtained from the same participants in different waves; thus, the consumption structure and diet quality for these respondents were correlated [1]. The cluster effects were controlled to eliminate such kind of influences. All statistical tests were two-tailed tests and with statistical significance level at *p* < 0.05. Data analysis in this study was completed by using the statistical/data analysis software package of STATA/MP 16.0.

## 3. Results

### 3.1. Descriptive Analysis

Table 2 presents the descriptive statistics of DQD score and the covariates of selected samples. There are 47.22% males and 52.78% females in the dataset. The rural residents and urban residents take up 67.37% and 32.63%, respectively. The average proportion of teens and elders in the households are 8.53% and 4.1%, respectively.

### 3.2. Dynamics of DQD

**Figure 2:**
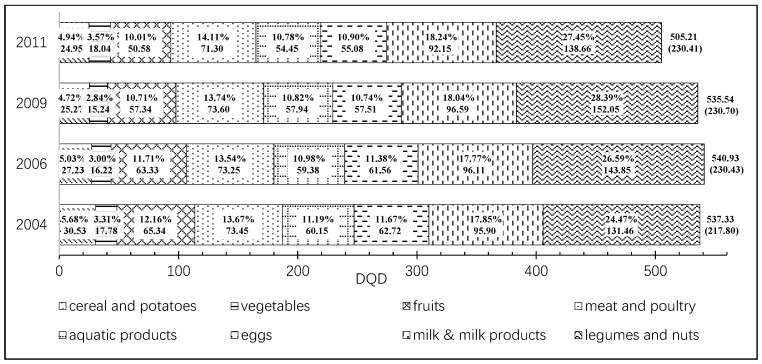
Dynamics of diet quality divergence Index (DQD) and its structure. DQD Change (2011–2004) = −32.11 (SD = 3.54), *p* < 0.001.

Figure 2 illustrates that the dietary divergence from the Chinese Food Pagoda 2016 generally declines over 2004–2011. The difference of DQD between 2004 and 2011 is −32.11 (*p* < 0.05), which implies that the diet quality improves in China during the period of 2004–2011. Figure 2 also depicts the structure of the DQD. In 2004–2011, the largest part of the DQD comes from legumes and nuts, and the proportion it takes also generally increases. The second largest part of the divergence derives from milk and milk products, and then followed by meat and poultry, fruits, eggs, and aquatic products. The absolute divergences for those food categories shrink from 2004 to 2011 which indicates the improvement of intakes for those foods. When it comes to the DQD from cereal and potatoes, it is also on a declining track over the period. Vegetables contribute the smallest DQD and remain stable during 2004–2011. Overall, the dynamics of the DQD present the transition of Chinese diet from staple cereals and vegetables to high protein and quality foods.

### 3.3. DQD for Different Subpopulation

**Figure 3:**
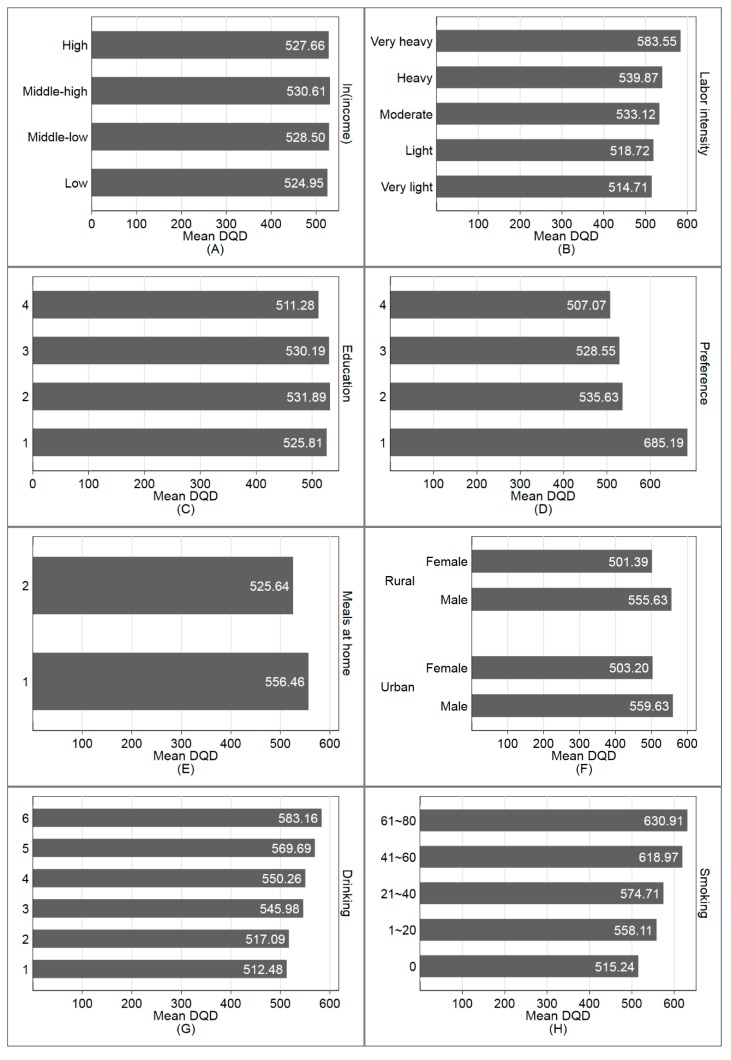

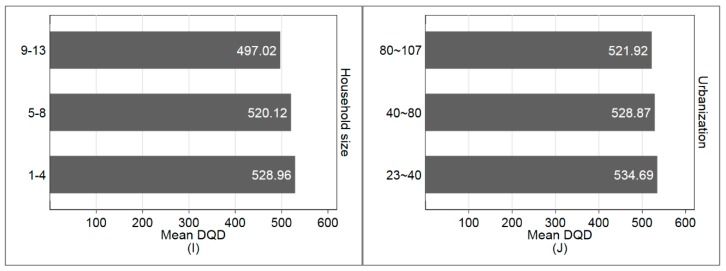
DQD for different subpopulations. (**A**) Across different quartile income groups; (**B**) across different labor intensity levels; (**C**) education: 1 = 0–6 years; 2 = 7–9 years; 3 = 10–12 years; 4 = more than 12 years; (**D**) healthy food preference index (the higher the healthier): 1 = 0–10; 2 = 11–15; 3 = 16–20; 4 = 21–25; (**E**) proportion of dining at home: 1= 0–50%, 2 = 50–100%; (**F**) Gender distribution across regions (rural vs urban); (**G**) Drinking frequency: 1 = no drinking; 2 = no more than once a month; 3 = once or twice a month; 4 = once or twice a week; 5 = 3–4 times a week; 6 = almost every day; (**H**) amount of cigarettes per day; (**I**) household size; (**J**) urbanization index.

The DQD varies for different population groups. Figure 3 shows that the DQD is slightly lower for groups with low/high income, and the dietary divergence increases as the labor intensity level grows. The medium education residents have a larger DQD than those with higher or lower education. When it comes to food preference index, people with strong healthy awareness and higher food preference index tend to get lower DQD which means that the healthy diet education would help the people to get healthy food preference and diet. The DQD for females is lower than that for males. Residents who drink and smoke tend to have higher DQD and poorer diet quality. Residents from larger families would get a lower DQD. People from a community with a higher urbanization index tend to have a lower DQD.

### 3.4. DQD and Its Influential Factors

#### 3.4.1. Multivariate Ordinary Least Squares Regression

In order to further explore impacts of diet quality influential factors on the DQD, a multivariate ordinary least squares regression with heteroscedasticity-robust standard errors was employed in the present study. Given the possible existence of interaction between income and food preference index, an interaction term of those two variables was adopted in the regression model. In addition, income may also be associated with external consumption environment [2]. Therefore, the interaction term of income and urbanization index was employed in the model. Some studies suggested that the influence of age on diet may be nonlinear [54,55], so age and squared age were adopted in the regression model [6]. The results were presented in Table 3.

The F-test of the model is statistically significant (*p* < 0.001). Most of the coefficients are statistically significant at least at the level of 10%, except for exercises time, sedentary time, proportion of elders (age > 64), dummy for 2006 and 2009. The average marginal effect of income, labor intensity, and drinking on DQD are positive and statistically significant (*p* < 0.01). Male and urban residents have higher DQD keeping other variables constant at means (*p* < 0.01). Smoking has significant positive (*p* < 0.05) impacts on DQD.

On the contrary, the impacts of urbanization, age, education, meals at home, household size, and proportion of teens (6–17) in the families on DQD are negative and statistically significant (*p* < 0.01). Healthy food preference also has a negative relationship with DQD (*p* < 0.01), as the preference for healthy food increases, the diet quality gets improved.

**Figure 4:**
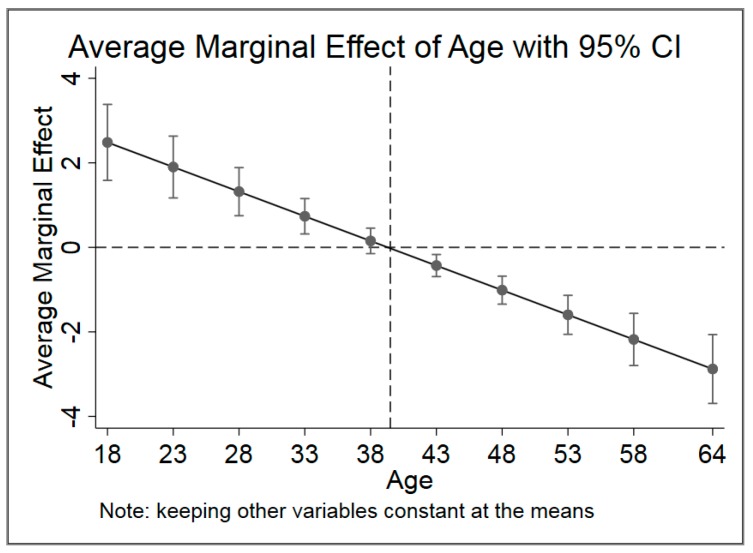
Average marginal effect of age with 95% CI.

As the coefficient of Age_square is negative, the relationship between age and DQD is an inverted U-shape. To shed light on the detailed relationship between age and DQD, the conditional marginal effects of age on DQD are estimated given other variables are fixed at means. Figure 4 reveals that as age increases, the marginal effect becomes negative after age about 40. 

#### 3.4.2. Multivariate Quantile Regressions

**Figure 5:**
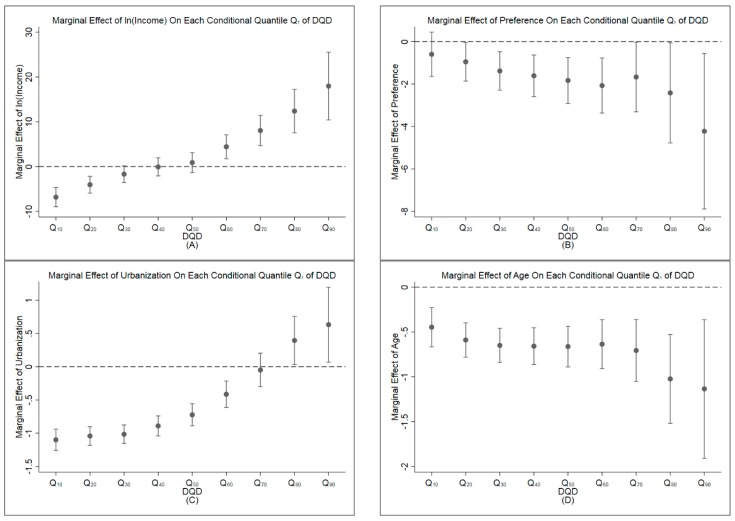
Marginal effect of co-variants on each DQD quantile with 95% CI. (**A**) The marginal effects of income on each DQD quantile; (**B**) the marginal effects of healthy food preference on each DQD quantile; (**C**) the marginal effects of urbanization on each DQD quantile; (**D**) the marginal effects of age on each DQD quantile.

In the multivariate regression model, the marginal effects of independent variables derived are assumed to be constant over the distribution of DQD. However, some literature pointed out that the relationships would be varied as the diet patterns change, so do the impacts of influential factors on DQD. To investigate the heterogeneous relationships between DQD and independent variables, quantile regressions were employed [37]. Therefore, from 1/10 quantile to 9/10 quantile, a total of nine quantile regressions were estimated and the results were reported in Table 4. The marginal effects of income, healthy food preference, urbanization, and age on each DQD quantile with 95% CI were predicted and the results were presented in Figure 5.

As the DQD quantile increases, the impacts of income on DQD increase. Figure 5 indicates that the marginal effect of income is negative in 1/10, 2/10, and 3/10 quantile regressions. In 4/10 and 5/10 quantile regressions, the marginal effects of income become positive, value 0 is in the 95% confidence interval. In 6/10, 7/10, 8/10, 9/10 quantile regressions, the marginal effects of income were positive and statistically significant (*p* < 0.05). Similar results are also found in impacts of urbanization on DQD, the marginal effects of urbanization shift from negative value to be positive as the DQD quantile augments. The marginal effects of healthy food preference decline slightly as DQD quantile increases. When it comes to the age, the results show that impacts of age are negative and statistically significant (*p* < 0.05) in all quantile regressions. 

### 3.5. Relationships between DQD and BMI 

According to the BMI, the respondents are categorized into four groups, including light weight, normal weight, overweight, and obesity. According to Table 5, the results of two samples t-test between normal group and obesity group implies that normal group has a lower DQD than obesity group (*p* < 0.05). The *t*-test between normal group and overweight group also indicates that normal group has a lower DQD than overweight group (*p* < 0.01). This suggests that DQD is a useful indicator for the dietary quality and health. 

## 4. Discussion

With the calculation of the divergence between the real diet intakes in the CHNS survey and the Chinese food pagoda 2016, namely DQD, this research explores the transition of the diet quality for Chinese residents and influential factors of DQD. Two samples *t*-test implies that the normal weight group significantly has a lower DQD (*p* < 0.05) than the obesity and overweight groups which means they get more balanced and healthy diet. That indicates DQD is one useful indicator to measure the diet quality for Chinese residents and to explain obesity and overweight.

### 4.1. Structural Changes of the DQD over 2004–2011

The DQD generally declines during 2004–2011, except for 2006. That means the overall Chinese diet quality improves over that period and Chinese residents get a more balanced diet. The results also indicate that the largest part of DQD is the category of legumes and nuts which takes up more than one quarter of DQD with the absolute value of over 100 in 2004–2011. According to the CHNS survey, the overconsumption of legumes and nuts is popular for Chinese residents. In addition, this result is also consistent with other studies [2,4]. The category of milk and milk products is the second largest source of Chinese DQD and the share it takes slightly increases over the period. The existing studies point out that Chinese generally intake insufficient milk [2,4,45]. The divergence value of meat and poultry declines slightly from 2009 to 2011 which implies the improvement of meat and poultry intakes in Chinese diet patterns.

According to the data from CHNS, the divergence values of fruits and eggs decrease. Even though the share of both food categories shrinks over the period, they are still bigger than 10% in 2011. That suggests the consumptions of fruits and eggs are lower than the recommendations. When it comes to cereal and potatoes, the divergence declines during 2004–2011 that is consistent with the existing study [4]. Many studies point out that the consumption of cereal decreases as income increases in China [35]. Vegetables group is the smallest part in DQD which is consistent with the vegetable dominated Chinese diet patterns. 

### 4.2. The Influencial Factors of DQD

A large body of literature suggests that income is an important influential factor of food consumption [35,37,38]. The average marginal effect of income in the multivariate regression on DQD is positive. Increasing income does not necessarily make the DQD smaller and improve the diet quality as we expected, instead, it enlarges the divergence of the diet patterns from the recommendations of Chinese food guideline which is consistent with the findings in the existence studies [1,27,56,57]. However, some literature also points out that income growth would improve the diet quality [49,58]. The relationships between DQD and income may change over different distributions of DQD. Therefore, to shed light on the detailed impacts of income on DQD, quantile regressions were adopted in this study. In 1/10, 2/10, and 3/10 quantile regressions, the marginal effects of income on DQD are negative and statistically significant (*p* < 0.05) that means the diet quality gets better as income grows. In 4/10 and 5/10 quantile regressions, the marginal effects of income are slightly positive when the confidence interval covers 0. In 6/10, 7/10, 8/10, 9/10 quantile regressions, the marginal effects of income on DQD become positive. These results reveal the nonlinear relationship between income and diet quality—DQD [35,36,37]. For Chinese residents with a small DQD and healthier diet, increasing income will help them to decrease the divergence between real dietary intakes and dietary guidelines keeping other factors constant at the same time. However, for those who have the DQD above the median, the raising income would enlarge the DQD and make diet quality worse. One possible explanation is that rising income would promote the consumption of some food which are not affordable for residents with low DQD before and help them to reach a more balanced diet, and it may also lead the increasing awareness of healthy food preference and diet [5,35]. Conversely, residents with high DQD usually hold unhealthy food preference, income growth may result in the increase of unhealthy food consumption (e.g., high calorie density food) [37].

The marginal effect of urbanization in multivariate regression is negative and statistically significant, which implies that development of urbanization could help Chinese residents to reduce their DQD and to get higher dietary quality. This result is consistent with the study from Huang, Hui, Xu, and Health [1]. However, some studies argue that urbanization may allow residents to consume more high-calorie foods and make their diet unbalanced [59,60]. The quantile regressions elaborate the nonlinear relationship between urbanization and DQD as the impacts of urbanization on different distribution of DQD change from negative to positive as the DQD quantile increases. In 1/10, 2/10, 3/10, 4/10, 5/10, 6/10 quantile regressions, the marginal effects of urbanization are negative and statistically significant (*p* < 0.05). It suggests that as urbanization improves, the Chinese residents with DQD lower than medium would get a lower DQD and more balanced diet. On the contrary, in 8/10 and 9/10 quantile regressions, the marginal effects of urbanization are positive and statistically significant (*p* < 0.05) which indicate that Chinese residents with upper quantile DQD cleave their diet from the guideline and make their DQD higher as the development of urbanization. Many researches point out that urbanization can improve the supply chain and food availability and residents with lower or medium DQD can consume more kinds of food which also makes their diet more balanced [1]. Reversely, residents with high DQD usually have unhealthy food preference and the urbanization process makes those with upper quantile DQD easily consume more unhealthy food (e.g., high caloric food) [61].

The average marginal effect of age in the multivariate regression implies that age is negatively correlated with DQD and statistically significant (*p* < 0.001). In all quantile regressions, the marginal effects of age are also negative and statistically significant (*p* < 0.05). The relationship between age and DQD is an inverted U-shape. As age increases from 18 to 64, the conditional marginal effect of age declines from positive to negative and the switch point is of 39.50 years old. Young people tend to pursue tasty food and sugared drinks that makes them easily get an unbalanced diet. However, when people get older, they usually pay more attention to healthy and keep a balanced diet in food consumption [1,4,58,62].

Labor intensity level is positively correlated with DQD and the groups with higher labor intensity tend to hold higher DQD. The marginal effects of labor intensity in multivariate regression and quantile regressions are positive which imply that the diet quality for those engaged in strong physical work would be worse. One reason might be that the strong physical workforces who are generally engaged in low-skilled physical activities (e.g., porter) need more calories. In addition, their diet may be dominated by carbohydrates (e.g., cereal and potatoes) and more likely get unbalanced [1].

Frequency of drinking alcohol is positively correlated with DQD. The marginal effects of drinking in multivariate regression and quantile regressions are positive which means the diet quality become worse as the frequency of drinking alcohol increases. The coefficient of smoking is positive in multivariate regression. As the number of cigarettes smoked per day increases, people would have a higher DQD. The marginal effects of smoking in multivariate regression and quantile regressions are also positive as smoking is highly likely associated with a lack of health awareness and unbalanced diet [48].

Education is negatively correlated with DQD in the regression. The average marginal effects of education in multivariate regression and quantile regressions are negative and statistically significant (*p* < 0.01). It is reasonable that people with higher education would get more information about dietary knowledge and pay more attention to diet balance [4,54]. The proportion of dining at home is negatively correlated with DQD. Chinese residents prefer to eat more food especially meat when dining out due to the traditional custom [4]. The household size is negatively correlated with DQD that is consistent with the Chinese Dietary Guidelines 2016 [44]. Larger families may purchase more types of food and help the family members get a balanced diet. The proportion of teens (aged 6–17) in the household is negatively correlated with DQD as Chinese parents generally pay more attention to the balance of dietary intakes for their adolescent children.

The results also indicate that females have lower DQD than males that is consistent with the existing studies [1,54]. Compared with males, females may have better dietary habits and pay more attention to keep a balanced diet. In addition, we find that the urban residents have higher DQD than the rural residents, probably because of the residents from urban have higher income, better food availability, and unhealthy food preference. 

### 4.3. The Impacts of DQD on BMI

Body mass index (BMI) is widely used to measure general obesity for adults. The results indicate that the increasing BMI usually accompanies the DQD growth. As the existence of divergences between real food intake and the Chinese food guideline, people with higher DQD are highly likely to get obesity and be overweight. This suggests that DQD is a useful indicator for the dietary quality and health. It also reveals the importance of nutrition education, especially the knowledge of healthy food and balanced diet which would greatly improve the diet quality and health for Chinese.

### 4.4. Limitations

There are several limitations for this study. First, oil and salt are not considered in the present study due to the data availability. In addition, many processed foods are not included in the Food Composition Table as the specific ingredients are not clear, snacks which are consumed other than three main meals (breakfast, lunch, and dinner) are also excluded [45]. Second, even though CHNS have put great efforts into the survey and data quality, it may still suffer from bias of dietary intakes in a 24 h recall method. Third, the mean of three days consumption data may not completely represent daily diet due to accidental factors (e.g., some foods were not consumed accidentally during the survey period) and seasonal variance in food demand. Fortunately, CHNS are conducted in autumn, which may minimize the variation of food availability [10]. Fourth, DQD measures the absolute value of the divergence between the real dietary intake and Chinese Food Pagoda 2016, which means DQD is scalar. It is necessary to check the actual food intake to determine whether the divergence is caused by over-intake or under-intake of food. Fifth, the present study adopted the CFP 2016 to measure the diet quality, and the results are only applicable to the Chinese adults, excluding the groups of infants, children, adolescents, pregnant women, breastfeeding women, and old people [44]. Sixth, due to the complexity of consumption and the availability of data, this study only selected some major factors and may get some missing variables. Further research with more detailed data is needed in the future. 

## 5. Conclusions

As for the rapid economy growth, Chinese are undergoing a fast nutrition transition and their traditional diet also turns into patterns associated with high quality and calorie and fat density foods. Those changes have strong impacts on national health. This study proposed the Chinese diet quality measurement—DQD to illustrate the transition of diet quality and its important influential factors. With the data from CHNS in 2004, 2006, 2009, and 2011, we composed the DQD for Chinese residents. The results indicate that the DQD generally declined over the period from 2004 to 2011. 

When it comes to the structure of the DQD, the biggest part of the divergence came from over-consumption of legumes and nuts, followed by under-consumption of milk and milk products. The category of meat and poultry was the third largest source of DQD. In addition, Chinese residents need to control the consumption of meat and poultry in the future according to the guideline. The divergences of fruits, aquatic products, and eggs decreased during 2004–2011 as Chinese residents are consuming more and more these products nowadays and in the future. The divergences of vegetables were the smallest due to the traditional Chinese diet.

The present study also carried out the DQD for different subpopulations, and the results reveal that people with poor diet quality are more likely to get increasing DQD and deteriorating diet quality. Females and urban residents have higher diet quality than their counterparts. This study also finds that impacts of income on diet quality are heterogeneous between different populations. Rising income is more likely to help people with higher diet quality to reach a balanced diet, while the population with lower diet quality may get bigger DQD and worse diet due to the rising income. The relationship between urbanization and diet quality is nonlinear as well. Urbanization could help Chinese residents with higher diet quality to reach a balanced diet and make the diet quality worse for those with high QDQ. We also find that healthy food preference, education, dining at home, household size, proportions of teens (6–17), and elders (over 64) in the families are positively correlated with Chinese residents’ diet quality. However, labor intensity, frequency of drinking alcohol and smoking have negative impacts on diet quality which makes the DQD become bigger. 

The results also indicate that higher DQD is associated with increasing risks of overweight/obesity which implies DQD is a useful index for the diet quality and health. It also suggests the importance of nutrition education for the Chinese to get a balanced diet and to keep a health body. 

All in all, to improve Chinese residents’ diet quality and health in the future, we suggest that national healthy policies should pay more attention to education on healthy food and nutrition for Chinese and help them to get healthy food preferences. It is also necessary to focus on populations with poor diet and adopt strict measures to control the bad habits, such as drinking alcohol and smoking.

## Figures and Tables

**Table 1:** The range of daily recommended intakes in Chinese food pagoda (CFP) 2016 and the corresponding ingredient code in China Food Composition Table (CFCT) 2002/2004.

Category	Food Group	Recommended Intake(g/d)	China Food Composition Table CFCT 2002/2004 (Ingredient Code)
1	Cereal and Potatoes	(250, 400)	(11.101, 22.203)
2	Fruits	(200, 350)	(61.101, 66.206)
3	Vegetables	(300, 500)	(41.101, 52.011)
4	Eggs	(40, 50)	(111.101, 114.201)
5	Aquatic Products	(40, 75)	(121.101, 129.302)
6	Meat and Poultry	(40, 75)	(81.101, 99.004)
7	Legumes and Nuts	(25, 35)	(31.101, 39.902), (71.001, 72.026)
8	Milk & Milk Products	300+	(101.101, 109.006)

Source: Chinese Dietary Guidelines 2016, China Food Composition Table 2002/2004.

**Table 2:** Descriptive statistics of selected variables (n = 30,626).

Variable	Description	Mean	SD ^1^
DQD	Divergence between real food consumption and CFP 2016 (%)	527.93	228.10
ln(income)	Log per capital income (ln(Yuan/year/capita))	8.97	1.08
Preference ^2^	Sum of preferences for food	17.89	2.13
Urbanization ^3^	Urbanization index	66.60	19.97
Region	Dummy for urban = 1 and rural = 0	0.33	0.47
Urban	The proportion of urban resident (%)	32.63	0.00
Rural	The proportion of rural resident (%)	67.37	0.00
Age	The age of the respondent (years)	44.83	11.73
Gender	Dummy for male = 1 and female = 0	0.47	0.50
Male	The proportion of male (%)	47.22	0.00
Female	The proportion of female (%)	52.78	0.00
Labor intensity ^4^	Level of labor intensity (level)	2.64	1.21
Education	Years of regular school education (years)	8.34	3.93
Meals at home	Proportion of dining at home per day (%)	2.59	0.66
Drinking ^5^	Frequency of drinking alcohol (level)	2.12	1.75
Smoking	Number of cigarettes consumed per day	4.73	9.01
Exercise	Exercises time per day (minutes)	14.21	54.82
Sedentary	Sedentary activities time per day (hours)	5.75	4.10
Household size	Number of family members (persons)	3.74	1.48
Teens (aged 6–17)	Proportion of teens aged 6–17 in the family (%)	8.53	14.09
Elders (over 64)	Proportion of elders over 64 in the family (%)	4.10	11.23
Y2006	Dummy variable for 2006 (%)	22.51	0.00
Y2009	Dummy variable for 2009 (%)	23.71	0.00
Y2011	Dummy variable for 2011 (%)	30.47	0.00

^1^ SD: Standard deviation; ^2^ The sum of preferences for five food categories, including healthy foods (i.e., fruits and vegetables) and unhealthy foods (i.e., fast food, salty snacks, soft drinks, and sugared fruit drinks), on a five-point Likert scale, the higher preference index indicates a healthier food preference; ^3^ Defined by a multidimensional 12-component urbanization index, including the population density, physical, social, cultural, and economic environment; ^4^ Labor intensity levels: 1 = very light physical activity, working in a sitting position; 2 = light physical activity, working in a standing position; 3 = moderate physical activity; 4 = heavy physical activity; and 5 = very heavy physical activity; ^5^ Drinking: 1 = no drinking; 2 = no more than once a month; 3 = once or twice a month; 4 = once or twice a week; 5 = 3–4 times a week; 6 = almost every day. Source: Calculated by the authors.

**Table 3:** Multivariate ordinary least squares regression (n = 30,626).

DQD	Coefficient	Robust SE	95% CI of Coef.	Marginal Effect	SE	95% CI of Marginal Effect
ln(income)	30.579 ***	10.299	(10.393, 50.766)	5.120 ***	1.268	(2.635, 7.605)
Preference	14.572 ***	5.235	(4.310, 24.834)	−1.759 ***	0.669	(−3.071, −0.447)
Urbanization	−1.354 ***	0.512	(−2.358, −0.349)	−0.395 ***	0.099	(−0.588, −0.201)
ln(income)*Preference	−1.821 ***	0.581	(−2.960, −0.682)			
ln(income)*Urbanization	0.107 *	0.059	(−0.008, 0.222)			
Region	26.392 ***	3.906	(18.736, 34.048)			
Age	4.582 ***	0.776	(3.061, 6.104)	−0.644 ***	0.141	(−0.919, −0.368)
Age_square	−0.058 ***	0.009	(−0.076, −0.041)			
Gender	38.468 ***	3.462	(31.681, 45.254)			
Labor intensity	5.259 ***	1.401	(2.512, 8.005)			
Education	−2.368 ***	0.437	(−3.224, −1.512)			
Meals at home	−9.614 ***	2.341	(−14.203, −5.025)			
Drinking	6.886 ***	0.977	(4.971, 8.801)			
Smoking	0.421 **	0.189	(0.051, 0.791)			
Exercise	0.025	0.026	(−0.025, 0.076)			
Sedentary	0.308	0.361	(−0.399, 1.016)			
Household size	−3.381 ***	0.987	(−5.315, −1.447)			
Teens (aged 6–17)	−0.655 ***	0.091	(−0.833, −0.476)			
Elders (over 64)	−0.181	0.118	(−0.412, 0.051)			
Y2006	4.122	3.782	(−3.292, 11.536)			
Y2009	−0.283	3.887	(−7.902, 7.336)			
Y2011	−30.888 ***	3.837	(−38.409, −23.367)			
Constant	243.807 ***	94.032	(59.500, 428.113)			
F (22, 30,603)	38.95					
*p* value > F	< 0.001					

Note: 1. SE: Standard deviation; 2. levels of statistical significance: *** 1%, ** 5%, * 10%; 3. the marginal effects are calculated with the coefficients of both the variables and interaction terms, keeping other variables constant at the means.

**Table 4:** Multivariate quantile regressions (n = 30,626).

Quantile	QR_10	QR_20	QR_30	QR_40	QR_50	QR_60	QR_70	QR_80	QR_90
ln(income)	–6.505	5.288	14.170 **	17.588 **	32.112 ***	27.960 ***	28.994 **	48.398 ***	62.441 **
	(8.317)	(7.260)	(7.220)	(7.809)	(8.619)	(10.337)	(13.095)	(18.807)	(29.244)
Preference	2.201	6.109 *	9.544 ***	9.854 **	15.862 ***	13.273 ***	13.440 **	20.332 **	20.184
	(4.128)	(3.604)	(3.583)	(3.876)	(4.278)	(5.130)	(6.499)	(9.334)	(14.515)
Urbanization	–1.808 ***	–1.682 ***	–1.816 ***	–1.592 ***	–1.272 ***	–1.367 **	–1.287 *	–0.867	0.064
	(0.457)	(0.399)	(0.397)	(0.429)	(0.473)	(0.568)	(0.719)	(1.033)	(1.606)
ln(income) * Preference	–0.312	–0.787 **	–1.218 ***	–1.278 ***	–1.972 ***	–1.711 ***	–1.684 **	–2.536 **	–2.721 *
	(0.452)	(0.394)	(0.392)	(0.424)	(0.468)	(0.561)	(0.711)	(1.022)	(1.589)
ln(income) * Urbanization	0.079	0.071	0.089 **	0.078	0.061	0.106 *	0.138 *	0.141	0.063
	(0.051)	(0.045)	(0.044)	(0.048)	(0.053)	(0.063)	(0.080)	(0.115)	(0.180)
Region	10.854 ***	15.688 ***	19.343 ***	21.634 ***	22.624 ***	21.142 ***	25.839 ***	34.378 ***	49.154 ***
	(3.024)	(2.640)	(2.625)	(2.840)	(3.134)	(3.759)	(4.762)	(6.839)	(10.634)
Age	1.703 ***	1.829 ***	2.621 ***	3.040 ***	3.858 ***	4.304 ***	4.728 ***	6.110 ***	11.897 ***
	(0.654)	(0.571)	(0.568)	(0.614)	(0.678)	(0.813)	(1.030)	(1.479)	(2.299)
Age_square	–0.024 ***	–0.027 ***	–0.036 ***	–0.041 ***	–0.050 ***	–0.055 ***	–0.061 ***	–0.080 ***	–0.145 ***
	(0.008)	(0.007)	(0.007)	(0.007)	(0.008)	(0.009)	(0.012)	(0.017)	(0.027)
Gender	26.722 ***	25.936 ***	27.464 ***	31.401 ***	36.298 ***	41.473 ***	47.039 ***	54.189 ***	63.629 ***
	(2.744)	(2.395)	(2.382)	(2.576)	(2.843)	(3.410)	(4.320)	(6.204)	(9.647)
Labor intensity	4.375 ***	4.186 ***	4.322 ***	4.742 ***	4.921 ***	5.848 ***	7.327 ***	8.360 ***	4.811
	(1.164)	(1.016)	(1.011)	(1.093)	(1.207)	(1.447)	(1.833)	(2.633)	(4.094)
Education	–2.460 ***	–2.473 ***	–2.353 ***	–2.295 ***	–2.028 ***	–2.033 ***	–2.068 ***	–2.775 ***	–3.500 ***
	(0.349)	(0.304)	(0.303)	(0.327)	(0.361)	(0.433)	(0.549)	(0.788)	(1.226)
Meals at home	–1.667	0.579	–2.352 *	–3.009 **	–5.038 ***	–5.477 ***	–9.017 ***	–10.606 ***	–35.716 ***
	(1.635)	(1.427)	(1.419)	(1.535)	(1.694)	(2.032)	(2.574)	(3.697)	(5.748)
Drinking	0.005	1.988 ***	2.889 ***	3.793 ***	5.425 ***	6.025 ***	8.620 ***	14.036 ***	15.066 ***
	(0.728)	(0.636)	(0.632)	(0.684)	(0.755)	(0.905)	(1.147)	(1.647)	(2.561)
Smoking	0.288 **	0.330 ***	0.537 ***	0.412 ***	0.310 **	0.278	0.241	–0.059	–0.135
	(0.139)	(0.121)	(0.120)	(0.130)	(0.144)	(0.172)	(0.218)	(0.313)	(0.487)
Exercise	–0.023	–0.015	0.007	0.003	0.001	0.033	0.056 *	0.101 **	0.045
	(0.020)	(0.017)	(0.017)	(0.018)	(0.020)	(0.024)	(0.031)	(0.044)	(0.069)
Sedentary	–0.566 **	–0.583 **	–0.463 *	–0.196	–0.065	0.177	0.618	1.266 **	1.843 *
	(0.279)	(0.243)	(0.242)	(0.261)	(0.289)	(0.346)	(0.438)	(0.630)	(0.979)
Household size	–0.776	–1.505 **	–1.699 ***	–1.802 **	–2.879 ***	–3.159 ***	–4.589 ***	–6.344 ***	–11.539 ***
	(0.764)	(0.667)	(0.663)	(0.717)	(0.792)	(0.950)	(1.203)	(1.728)	(2.686)
Teens (aged 6–17)	–0.122	–0.243 ***	–0.306 ***	–0.295 ***	–0.357 ***	–0.439 ***	–0.566 ***	–0.872 ***	–1.373 ***
	(0.078)	(0.068)	(0.068)	(0.073)	(0.081)	(0.097)	(0.123)	(0.177)	(0.275)
Elders (over 64)	–0.215 **	–0.167 **	–0.176 **	–0.193 **	–0.118	–0.170	–0.126	–0.273	–0.047
	(0.093)	(0.081)	(0.081)	(0.087)	(0.096)	(0.115)	(0.146)	(0.210)	(0.327)
Y2006	–1.770	–0.133	0.332	–1.307	–1.613	–2.065	0.628	4.064	18.097 *
	(3.083)	(2.691)	(2.676)	(2.894)	(3.195)	(3.831)	(4.854)	(6.971)	(10.839)
Y2009	–3.494	–2.595	–1.007	–2.821	–2.422	–1.554	–2.777	0.932	8.897
	(3.177)	(2.773)	(2.757)	(2.983)	(3.292)	(3.948)	(5.001)	(7.183)	(11.169)
Y2011	–16.319 ***	–18.413 ***	–20.949 ***	–24.889 ***	–26.115 ***	–29.529 ***	–34.920 ***	–42.179 ***	–47.807 ***
	(3.101)	(2.706)	(2.691)	(2.911)	(3.213)	(3.853)	(4.881)	(7.011)	(10.901)
Constant	450.487 ***	388.146 ***	338.778 ***	327.235 ***	209.661 ***	257.886 ***	263.140 **	127.170	124.071
	(76.772)	(67.015)	(66.642)	(72.080)	(79.558)	(95.409)	(120.871)	(173.591)	(269.926)

Note: 1. Standard errors are provided in parentheses. 2. levels of statistical significance: *** 1%, ** 5%, * 10%.

**Table 5:** The relationships between DQD and body mass index (BMI).

Groups	Number of obs.	Mean DQD	Standard Deviation	Difference from Normal	*p*-Value of *t*-Test
Light	1509	512.46	202.64		
Normal	16781	525.13	221.67		
Overweight	9418	532.85	234.5	−7.72	<0.01
Obesity	2918	536.17	254.01	−11.04	<0.05
